# Foundations of the Quaternion Quantum Mechanics

**DOI:** 10.3390/e22121424

**Published:** 2020-12-17

**Authors:** Marek Danielewski, Lucjan Sapa

**Affiliations:** 1 Faculty of Materials Science & Ceramics, AGH UST, Mickiewicza 30, 30-059 Kraków, Poland; 2 Faculty of Applied Mathematics, AGH UST, Mickiewicza 30, 30-059 Kraków, Poland; sapa@agh.edu.pl

**Keywords:** relativistic quaternion quantum mechanics, Cauchy-elastic solid, Schrödinger and Poisson equations, quaternions, Klein–Gordon equation

## Abstract

We show that quaternion quantum mechanics has well-founded mathematical roots and can be derived from the model of the elastic continuum by French mathematician Augustin Cauchy, i.e., it can be regarded as representing the physical reality of elastic continuum. Starting from the Cauchy theory (classical balance equations for isotropic Cauchy-elastic material) and using the Hamilton quaternion algebra, we present a rigorous derivation of the quaternion form of the non- and relativistic wave equations. The family of the wave equations and the Poisson equation are a straightforward consequence of the quaternion representation of the Cauchy model of the elastic continuum. This is the most general kind of quantum mechanics possessing the same kind of calculus of assertions as conventional quantum mechanics. The problem of the Schrödinger equation, where imaginary ‘*i*’ should emerge, is solved. This interpretation is a serious attempt to describe the ontology of quantum mechanics, and demonstrates that, besides Bohmian mechanics, the complete ontological interpretations of quantum theory exists. The model can be generalized and falsified. To ensure this theory to be true, we specified problems, allowing exposing its falsity.

## 1. Introduction

Quantum mechanics is considered to be an irreducibly statistical theory, as a result unable to predict the behavior of individual processes. On the other hand, it has been increasingly used, with stunning success, to gain control over individual objects on an atomic scale. This situation motivates the research into the foundations, leading to a variety of approaches towards an adequate theoretical justification of individual phenomena. It is not agreed whether such an interpretation requires a modification of the standard quantum formalism or whether it can be achieved within that formalism [1]. The first possibility of an ontological, i.e., realist, quantum mechanics was introduced with the original de Broglie–Bohm theory.

Quaternion quantum mechanics, QQM, presented by us is ontic in the sense that it answers the central question of interpretation of quantum mechanics. It is directly related to being (the Cauchy elastic continuum) as well as to the basic categories of being and their relations. The main concepts of quaternion quantum mechanics (QQM) for both the general and mathematical audience are shown. The ideas coming from the quantum theory remain almost completely unfamiliar to most mathematicians who found it difficult to absorb physical ideas, mainly because of the absence of clear definitions and statements of the concepts involved. This paper attempts to overcome some of these gaps in communication. The subject is incredibly huge, and selections are unavoidable.

Quantum mechanics, where we are. The quantum mechanics foundation remains a subject of discussion ever since it was presented in the 1920s. An instantaneous process of the wave-function collapse doesn’t fit together with the speed of light limit in Special Relativity. This is the “spooky action” that irritated Einstein so much about quantum mechanics. Present explanations base on assumption that collapse incompatibility has no observable consequences and it’s philosophically permissible. However, the problem stays when one asks what happens with the mass and energy of a particle when its wave-function collapses. Notice that the instantaneous jump is not expected in General Relativity and the unclear “string theory” doesn’t help. 

The Copenhagen interpretation represents the main-stream view; yet recent years witness revived interest in the alternative deterministic interpretation, pioneered by Madelung, de Broglie, and Bohm. David Bohm and Basil Hiley developed an interpretation of quantum mechanics which gives a clear and intuitive interpretation of its meaning with no need of assuming a fundamental role for the human observer [2]. Their ontological interpretation means the causal interpretation of quantum mechanics and is a refinement and completion of de Broglie’s 1926 pilot-wave model of non-relativistic quantum theory rediscovered by Bohm in 1952 [3]. This deterministic interpretation is still considered as basically equivalent to the Copenhagen orthodox understanding. It is noteworthy that high-level scientists and even S. Weinberg do not consider it important to mention the unclear issues of the Copenhagen interpretation, the topics that motivated Bohm. The importance of the Bohm approach, i.e., the fact that it consistently solves the measurement problem and allows the classical description of macroscopic objects, is frequently ignored.

John S Bell [4] has given a tremendous input to our understanding of reality by making clear that nonlocal features characterize natural processes. Even so, he also was expressing dissatisfaction with the conceptual and logical status of the theory and has indicated only two possible ways to solve problem [5]:

“Either the wavefunction, as given by the Schrödinger equation, is not everything, or it is not right.”

Bell’s immense prestige has pushed many physicists to reexamine old problems. We are now observing a comeback of the imprecise and unprofessional thinking which have characterized the debate in the second quarter of the 20th century. Particularly noteworthy is the domination of an incorrect, accepted by many scientists interpretation concerning the real meaning of Bell’s theorem [6]. What really matters is the fact that the derivation of Bell’s inequality in no way whatsoever needs an assumption of realism. In spite of this fundamental fact, which everybody can verify by going carefully through the proof, a large part of the scientific community shares the wrong opinion that realism is among the basic assumption needed for the derivation of Bell’s result. Bell himself has stressed this aspect and commented [7] that it is extremely difficult to eradicate this prejudice:

“My own first paper [4] on this subject starts with a summary of the EPR argument from locality to deterministic hidden variables. But the commentators have almost universally reported that it begins with deterministic hidden variables.”

Bell’s inequality has nothing to do with realism, it straightforward identifies that what quantum phenomena impose to us is to accept the inescapable fact that natural processes involving entangled states of composite and far-away systems turn out to be unavoidably non-local. 

Where we are today? There is a widely known remark by Richard Feynman in 1964:

“It is safe to say that no one understands quantum mechanics”

[8]

and Murray Gell-Mann statement in his lecture at the 1976 Nobel Conference that

“Niels Bohr brainwashed the whole generation of theorists into thinking that the job (of finding an interpretation of quantum mechanics) was done 50 years ago”

[9]

are both actual. The deterministic interpretation that was considered as basically equivalent to the Copenhagen orthodox understanding is not in use. It was reasoned that since this theory is empirically indistinguishable from the standard theory, it should be considered an example of “bad science”. S Weinberg in a letter to S Goldstein [10,11] explicitly expressed such a way of thinking:

“At the regular weekly luncheon meeting today of our Theory Group, I asked my colleagues what they think of Bohm’s version of quantum mechanics. The answers were pretty uniform and much what I would have said myself. First, as we understand it, Bohm’s quantum mechanics uses the same formalism as ordinary quantum mechanics, including a wavefunction that satisfies the Schrödinger equation, but adds an extra element, the particle trajectory. The predictions of the theory are the same as for ordinary quantum mechanics, so, there seems little point in the extra complication, except to satisfy some a priori ideas about what a physical theory should be like… In any case… we are all too busy with our own work to spend time on something that doesn’t seem likely to help us make progress with our real problems.”

Here, it is important to mention that the predictive equivalence of the two theories is no more obvious [12]. It is safe to say that none of the existing interpretations and modifications of quantum mechanics truly solve the problem of how to derive it from fundamental laws, is it ontic, etc.

Quaternion quantum mechanics today. The first suggestion of quaternion quantum mechanics appears in a footnote of Birkhoff and J. von Neumann 1936 paper [13]. They suggest, in particular, that the physically significant statements in quantum mechanics actually constitute a sort of projective geometry, while the physically significant statements concerning a given system in classical dynamics constitute the Boolean algebra. This indicates that quantum mechanics has greater logical consistency than classical mechanics; a conclusion validated by the impossibility in general of measuring different quantities independently.

Yang has pointed out [14] that it is always possible to represent the pure states of a system of “general quantum mechanics” by rays in a vector space in a one-to-one manner, and for this, it is necessary and sufficient to employ suitable orthogonal vector subspaces of some Hilbert spaces, *H*, over the following fields of numbers:

R, the real numbers,
C, the complex numbers,
Q, the quaternions.

This result suggests that it is not necessary to go beyond the three possibilities R,C and Q for the representation of general quantum mechanics (the Hurwitz Theorem states that the real numbers R, complexes C, quaternions Q and octonions O are the only normed division algebras over the real numbers). A quantum mechanics coefficients assuming values that are quaternionic was proposed by Finkelstein et al. [15]. It was shown that a quaternion calculus exists that they called general quantum mechanics (as distinguished from complex quantum mechanics) and it is always possible to represent pure states of a system of general quantum mechanics by rays in a vector space over the quaternions, but not so over the real and complex numbers. These authors use Stone’s theorem to explain the imaginary number “*i*” in the Schrödinger equation however, the central problem of finding feasible dynamics for quaternionic quantum theory has remained unsolved. More recently, the global effects in quaternionic quantum field theory [16] were applied to analyze the experimental status of quaternionic quantum mechanics [17].

The algebra of complex numbers, quaternions and octonions play also an important role in the physical interpretation of the standard model for electroweak interactions and quantum chromodynamics [18]. It is known that certain nonlinear Schrödinger (NLS) equations, in one or more space dimensions, possess space-localized solutions ψ=ψ(t,x), e.g., solitons in the one-dimensional case. Bodurov has shown that the same result is valid for a large class of complex nonlinear wave equations and NLS equations [19,20]. Białynicki-Birula and Mycielski have found that NLS equation admits closed-form space localized solutions (gaussons) [21]. They have shown also that *“…in every electromagnetic field, sufficiently small gaussons move like classical particles*”. Weng adopted the complex quaternion and octonion to formulate the field equations for electromagnetic and gravitational fields. The results reveal that the quaternion space is appropriate to describe the gravitational features [22]. Consistent with QQM are also the Three Wave Hypothesis by Horodecki that is based on de Broglie’s particle-wave duality and the assumption of covariant æther [23,24]. Recently Gantner demonstrated the equivalence of complex and quaternionic quantum mechanics [25].

The Klein–Gordon and Schrödinger equations are important tools for describing quantum mechanics, respectively relativistic and non-relativistic. Their stationary versions allow us to find the values of quantized energy as the eigenvalues of self-adjoint operators on the Hilbert spaces. Adler studied downgraded quaternion-imaginary Lagrangian and showed that a quaternionic quantum field theory can be formulated when the numbers of bosonic and fermionic degrees of freedom are equal [26]. More recently he studied the quaternionic projective group representations and so-called trace dynamics in Hilbert spaces [27,28]. His idea of the trace dynamics relies on using a variational principle based on a Lagrangian constructed as a trace of noncommuting operator variables, making systematic use of cyclic permutation under the trace operation. In our paper we construct a Lagrangian with the use of the Cauchy–Riemann operator, acting on quaternionic valued functions. Nottale’s contribution was the derivation of the physical and mathematical tools of quantum mechanics by using the bi-quaternion concept. His approach proposes an answer to the question of the origin of complex and bi-quaternionic numbers, and more generally of Clifford algebra in quantum mechanics [29]. Recently, a quaternionic commutator bracket was proposed by Arbab et al. [30].

Our quaternion Klein–Gordon and Schrödinger equations have much more physical information than their complex equivalents, i.e., make it a much richer theory. In this work we will derive the Schrödinger equation with the use of the variational calculus, minimalizing the suitable integral functional generated by our constructed Lagrangian. Further qualitative analysis of these quaternionic equations will be the subject of our future papers.

Summing up, the quaternion quantum mechanics has many new features which make it a much richer theory. It is caused generally by the noncommutativity of quaternion-valued wave functions. It has not yet been more fully developed mainly due to the problem of how to write the Schrödinger equation. Remains valid the remark by Lord Kelvin (alias William Thomson, who formulated the laws of thermodynamics) [31]:

“Quaternions came from Hamilton…and, though beautifully ingenious, have been an unmixed evil to those who have touched them in any way, including Clerk Maxwell.”

The Planck–Kleinert crystal. It was shown that quantum gravity effects modify Heisenberg’s uncertainty principle to a generalized uncertainty principle (GUP) [32]. The GUP-induced corrections to the Schrödinger equation, when applied to a non-relativistic particle in a one-dimensional box, led to the quantization of length and the result was interpreted as a signal of fundamental discreteness of space itself. Similarly, corrections to the Klein–Gordon and Dirac equations gave rise to length, area and volume quantizations, again indicative of the fundamentally grainy nature of space. Such an approach modifies all quantum mechanical Hamiltonians [33]. When applied to an elementary particle, it implies that space which confines it must be quantized. This suggests that space itself is discrete, and that all measurable lengths are quantized in units of a fundamental length (which can be the Planck length).

The original arguments to implement the classical mechanics equations in the field of wave mechanics were given by Kleinert [34]. Kleinert combined Planck scale approaches to Quantum Gravity (such as String Theory and Doubly Special Relativity, black hole physics) that predict a minimum measurable length. The building blocks of the Kleinert continuum (solid) are Planck particles, *m_P_*, that obey the laws of mass, momentum and energy conservation. Each particle exerts a short-range force at the Planck length. The Kleinert concept linked with the Cauchy model of the elastic continuum has been later analyzed with the arbitrary assumption of the complex potential field [35]. Recently, the Cauchy theory was rigorously combined with the Helmholtz decomposition of the vector field of deformations together with quaternion algebra [36] and such representation of the Cauchy equation of motion produced the Klein–Gordon wave equation [37].

In this paper, we present the fundamentals of the quaternion quantum theory, with the clear and precise specification of what the theory is basically about. The fundamental new results, explicitly the deformations represented in quaternion algebra and the family of waves in the elastic continuum are derived. 

The essentials of quaternion algebra are given in Section 2.1, and the Cauchy model of the elastic continuum in Section 2.2. Both can be omitted by the experienced reader. In Section 3 we present the rigorous derivation of the quaternion representation of the Cauchy deformation field that allows considering multiple forms of waves and standing waves in ideal elastic solid. The final result is the vast possibility of waveforms in the elastic continuum.

## 2. Methods

### 2.1. Essentials of the Quaternion Algebra

The algebra of quaternions, Q, owns all laws of algebra with unique properties [38]. The essential here are (1) the multiplication of quaternions that is noncommutative and (2) it allows quantifying twists and compression. In the next sections, we attempt to convince readers that quaternions are a physical reality. Not only helpful and convenient, the quaternions also allow entering and understanding the processes in continua, together with the wave mechanics. 

Hamilton tried for ten years to create the $R^{3}$ analog of the complex numbers. His unquestionable motivation was the physics of solids and liquids. Finally in 1843, while on a walk with his wife Helen, he realized that three distinct imaginary units are necessary. He carved a new idea on the Broom Bridge in Dublin, which today is immortalized by a commemorative plaque [39]. The beauty of quaternions was immediately recognized, James Clerk Maxwell in 1869 wrote [40]:

“*The invention of the calculus of quaternions is a step towards the knowledge of quantities related to space which can only be compared for its importance, with the invention of triple coordinates by Descartes. The ideas of this calculus are fitted to be of the greatest in all parts of science.*”

In spite of this, all Maxwell attempts to reformulate electromagnetism using quaternions were unsuccessful [31].

The simplified, trivial and unfortunately very common opinion tells that quaternions were invented as an extension to the complex numbers. Nowadays they are in mass use in the computation of rotations… in every computer graphics film studio. In the same way, the calculus of imaginary numbers by many is considered as the justified useful tool, by no means the physical reality. Not many scientists agree that reformulating basic principles in terms of quaternion algebra allows a deeper understanding of physics.

Our review of basic definitions and formulas of the quaternion numbers and functions is shortened and limited to those used in the paper [38]. In the original Hamilton notation, a quaternion is regarded as the sum of a real (scalar) and imaginary (vector) part: $\sigma =\sigma_{0}1+\hat{\phi }=\left[\sigma_{0},\hat{\phi }\right]\in Q$. An arbitrary quaternion σ∈Q can be written in terms of its basic components

(1) $$\sigma =\left(\sigma_{0},\phi_{1},\phi_{2},\phi_{3}\right)\in Q,$$

where the four $\sigma_{0},\phi_{i}$ coefficients are real. In (1) we introduced notation that is right and convenient in the case of an ideal elastic continuum where only the compression, $\sigma_{0}$, and twist (torsion) emerge, explicitly: $\sigma_{1}=\phi_{1}$, $\sigma_{2}=\phi_{2}\;\mathrm{and}\;\sigma_{3}=\phi_{3}$.

Rigorously, in the mathematical way, the quaternion algebra Q can be defined as follows. Let $R^{4}$ be the four-dimensional Euclidean vector space with the orthonormal basis $\left\{e_{0},e_{1},e_{2},e_{3}\right\}$, where $e_{0}=\left(1,0,0,0\right)$, $e_{1}=\left(0,1,0,0\right)$, $e_{2}=\left(0,0,1,0\right)$, $e_{3}=\left(0,0,0,1\right)$ and with the three-dimensional vector subspace $P=span\left\{e_{1},e_{2},e_{3}\right\}$. We introduce the multiplication law by the formula

(2) $$a\cdot b=\left(a_{0}b_{0}-\hat{a}\circ \hat{b}\right)e_{0}+\hat{a}\times \hat{b}+a_{0}\hat{b}+\hat{a}b_{0},$$

where $a=\sum_{i=0}^{3}a_{i}e_{i}$, $b=\sum_{i=0}^{3}b_{i}e_{i}\in R^{4}$, $\hat{a}=\sum_{i=1}^{3}a_{i}e_{i}$, $\hat{b}=\sum_{i=1}^{3}b_{i}e_{i}\in P$ and ∘, × mean the scalar and vector products in P, respectively:

$$\hat{a}\circ \hat{b}=\sum_{i=1}^{3}a_{i}b_{i},$$

$$\hat{a}\times \hat{b}=\det\left[\begin{array}{ccc} e_{1} & e_{2} & e_{3}\\ a_{1} & a_{2} & a_{3}\\ b_{1} & b_{2} & b_{3} \end{array}\right].$$

Note that these products are analogous as in $R^{3}$.

The multiplication given by (2) is noncommutative. The vector space $R^{4}$ with the multiplication (2) is a noncommutative algebra with unity usually denoted by Q and it is named the quaternion algebra. In practice, the following algebraical notation is useful: $e_{0}=1,\;e_{1}=i,\;e_{2}=j,\;e_{3}=k$. By the definition (2), the quaternion imaginary units obey the following relations:

(3) $$i^{2}=j^{2}=k^{2}=-1,\;ij=-ji=k,\;jk=-kj=i,\;ki=-ik=j.$$

Thus for an arbitrary quaternion σ∈Q, we can write shortly

(4) $$\sigma =\sigma_{0}1+\phi_{1}i+\phi_{2}j+\phi_{3}k=\sigma_{0}1+\hat{\phi }$$

A conjugate quaternion is defined as

(5) $$\sigma^{*}=\sigma_{0}1-\phi_{1}i-\phi_{2}j-\phi_{3}k=\sigma_{0}1-\hat{\phi }.$$

It follows immediately from (2)–(5) that $\sigma \cdot \sigma^{*}=\sigma^{*}\cdot \sigma =\sigma_{0}^{2}+\sum_{l=1}^{3}\phi_{l}^{2}$, and therefore the Euclidean norm

(6) $$\left\|\sigma \right\|=\sqrt{\sigma \cdot \sigma^{*}}.$$

Hence Q is a normed algebra.

Let $\Omega \subset R^{3}$ be a bounded set. The so-called Q-valued functions may be written as

(7) $$\sigma \left(x\right)=\sigma_{0}\left(x\right)1+\phi_{1}\left(x\right)i+\phi_{2}\left(x\right)j+\phi_{3}\left(x\right)k,\;x=\left(x_{1},x_{2},x_{3}\right)\in \Omega ,$$

where the functions $\sigma_{0}\left(x\right),\;\phi_{l}\left(x\right),\;l=1,2,3$ are real-valued. Properties such as continuity, differentiability, integrability and so on, which are ascribed to σ have to be possessed by all the components $\sigma_{0}\left(x\right),\;\phi_{1}\left(x\right),\;\phi_{2}\left(x\right),\;\phi_{3}\left(x\right)$. In this manner, the Banach, Hilbert and Sobolev spaces of Q-valued functions can be defined [38]. In the Hilbert space over Q,

(8) $$L^{2}\left(\Omega \right)=\left\{\sigma :\Omega \to Q|\int_{\Omega }\sigma_{0}^{2}dx<\infty ,\;\int_{\Omega }\phi_{l}^{2}dx<\infty ,\;l=1,2,3\right\}$$

we introduce the inner product as follows

(9) $$\langle \sigma_{1},\sigma_{2}\rangle =\int_{\Omega }\sigma_{1}^{*}\cdot \sigma_{2}dx,\;\sigma_{1}^{},\sigma_{2}\in L^{2}\left(\Omega \right).$$

In a similar way, we define the Sobolev spaces

(10) $$H^{k}\left(\Omega \right)=\left\{\sigma :\Omega \to Q|\sigma ,\sigma^{\left(1\right)},\ldots ,\sigma^{\left(k\right)}\in L^{2}\left(\Omega \right)\right\},\;k\in N.$$

The definition of self-adjoint operators acting on these spaces is analogous as in the real and complex cases. Moreover, the theories of analytic functions, distributions, Fourier series, Lebesgue measure, Gelfand triples, Laplace transform and many others on the vector space of Q-valued functions over Q can be defined in a standard way as in the real and complex cases with analogous properties.

Similarly, the functions σ(t,x) depending on time *t* may be considered.

We will use the Cauchy–Riemann operator *D* acting on the quaternion-valued functions

(11) $$D\sigma =\left(-\mathrm{div}\hat{\phi }\right)1+\mathrm{grad}\sigma_{0}+\mathrm{rot}\hat{\phi },\;\sigma =\sigma_{0}1+\hat{\phi },$$

where $\mathrm{grad}\;\sigma_{0}=\frac{\partial \sigma_{0}}{\partial x_{1}}i+\frac{\partial \sigma_{0}}{\partial x_{2}}j+\frac{\partial \sigma_{0}}{\partial x_{3}}k$, $\mathrm{div}\hat{\phi }=\frac{\partial \phi_{1}}{\partial x_{1}}+\frac{\partial \phi_{2}}{\partial x_{2}}+\frac{\partial \phi_{3}}{\partial x_{3}}$ and

$$\mathrm{rot}\hat{\phi }=\det\left[\begin{array}{ccc} i & j & k\\ \frac{\partial }{\partial x_{1}} & \frac{\partial }{\partial x_{2}} & \frac{\partial }{\partial x_{3}}\\ \phi_{1} & \phi_{2} & \phi_{3} \end{array}\right].$$

Under the constraint $\mathrm{div}\hat{\phi }=0$, *D* corresponds physically to the nabla operator ∇ in $R^{3}$:

(12) $$D\sigma =\mathrm{grad}\sigma_{0}+\mathrm{rot}\hat{\phi },\;\sigma =\sigma_{0}1+\hat{\phi }.$$

The exponent function has its trigonometrical representation

(13) $$e^{\sigma }=e^{\sigma_{0}}\left(\cos\left|\hat{\phi }\right|+\frac{\hat{\phi }}{\left|\hat{\phi }\right|}\sin\left|\hat{\phi }\right|\right),\sigma =\sigma_{0}1+\hat{\phi },$$

where *σ* is a Q-valued function.

**Remark** **1.** *Note that DDσ=−Δσ, thus Equation (12) links quaternion quantum mechanics to reality in $R^{3}$.*

**Remark** **2.** *In further parts we will identify a vector $\sigma_{0}1$ with a real number $\sigma_{0}$ so we will write for simplicity $\sigma =\sigma_{0}+\hat{\phi }$. Therefore Q can be treated as the generalized simple sum R⊕P.*

### 2.2. Cauchy Classical Theory of Elasticity

Cauchy finished the theory of the ideal elastic continuum in 1822 [41,42], right away Poisson [43] studied the elementary waves. In 1885 Neumann [44] gave the proof of the uniqueness of solutions of some boundary-initial value problems and before the end of the XIX-th century, a rigorous completeness proof was given by Duhem [45]. Cauchy theory is the first real attempt to construct a theory of elasticity using the continuum approach, where the macroscopic phenomena are described in terms of field variables [46,47]. Important here is that:

1. mathematical analysis of the various phenomena;
2. the Cauchy and to the same degree the majority of physical problems cannot be reduced to vectorial models (the vector product does not permit the formulation of algebra with unity, for example, the division operation is not defined [48]);
3. the Hamilton algebra of quaternions, Q, and Hamilton concept of the four-dimensional space allow combining Cauchy theory with the Helmholtz decomposition theorem.

In the following, we consider FCC structure, where the Poisson number ν = 0.25, $l_{P}$ denotes the dimension of the FCC elementary cell that consists of four interacting Planck particles showing the mass $m_{P}$.

1. The continuum is treated as a closed system occupying the constant volume $\Omega \subset R^{3}$.
2. The continuum density, $\rho_{P}$, is high and we consider the small deformation limit only, $l_{P}=const.$, thus the density changes are negligible and $\rho_{P}=4m_{P}/l_{P}^{3}=const.$
3. The small deformation limit implies invariant transverse wave velocity: $c=\sqrt{0.4Y/\rho_{P}}=const.$, where *Y* is the Young modulus [46], Equation (16).
4. In agreement with the Helmholtz decomposition theorem [46], every lattice deformation **u** can be expressed as a sum of compression and twist, $u=u_{0}+u_{\phi }$.
5. We will consider here the long evolution times, $t\gg t_{P}$, where $t_{P}$ is Planck time and the moving (translating) waves, υ, where υ is the velocity of the wave mass center as defined by Bodurov [19,20].

In this section, we do not consider the external fields. In such a continuum, the equation of motion relates to local acceleration due to the displacement with the field variables, the compression (divu) and twist (rotu)

(14) $$\frac{\partial^{2}u}{\partial t^{2}}=3c^{2}\mathrm{grad}\mathrm{div}u-c^{2}\mathrm{rot}\mathrm{rot}u.$$

From Equation (14), the energy per mass unit in the deformation field follows [46,49]

(15) $$e=\frac{\rho_{E}}{\rho_{P}}=\frac{1}{2}\dot{u}\circ \dot{u}+\frac{3}{2}c^{2}{\left(\mathrm{div}u\right)}^{2}+\frac{1}{2}c^{2}\mathrm{rot}u\circ \mathrm{rot}u,$$

where $\dot{u}=\partial u/\partial t$.

Equation (14) and relation (15) obey the Euler–Lagrange relation $\frac{\partial e}{\partial u}-\frac{d}{dt}\left(\frac{\partial e}{\partial \dot{u}}\right)=0$. It means that one can derive the vector equation of motion from the scalar relation of energy conservation and vice versa. The scalar relation (15) and the vector Equation (14) rule the deformation in the ideal elastic continua. By the Helmholtz decomposition theorem, every deformation can be expressed by the compression, $u_{0}$, and twist, $u_{\phi }$, and if **u** belongs to the $C^{3}$ class of functions then $u=u_{0}+u_{\phi }$, where $\mathrm{rot}u_{0}=0$ and $\mathrm{div}u_{\phi }=0$. Upon acting on Equation (14) by the divergence and rotation operators, we decompose it and get well known transverse and the longitudinal wave equations in the usual form $u_{tt}=k\Delta u$:

(16) $$\begin{array}{l} \mathrm{div}\left(\frac{\partial^{2}u}{\partial t^{2}}=3c^{2}\mathrm{grad}\mathrm{div}u-c^{2}\mathrm{rot}\mathrm{rot}u\right)\Rightarrow \frac{\partial^{2}}{\partial t^{2}}\mathrm{div}u_{0}=3c^{2}\Delta \mathrm{div}u_{0},\\ \mathrm{rot}\left(\frac{\partial^{2}u}{\partial t^{2}}=3c^{2}\mathrm{grad}\mathrm{div}u-c^{2}\mathrm{rot}\mathrm{rot}u\right)\overset{\Delta u=\nabla \left(\nabla \cdot u\right)-\nabla \times \left(\nabla \times u\right)}{\Rightarrow }\frac{\partial^{2}}{\partial t^{2}}\mathrm{rot}u_{\phi }=c^{2}\Delta \mathrm{rot}u_{\phi }. \end{array}$$

The Cauchy equation of motion combined with the Helmholtz decomposition theorem results in four uncoupled second-order scalar differential equations, “quattro cluster”, and implies the transverse and longitudinal waves in the Cauchy elastic solid. Note that these equations remain coupled with the relation of the energy density, Equation (15). In the next sections, using the quaternion representation of the Cauchy elastic continuum, the plentiful forms of the wave equations are derived.

## 3. Results

### 3.1. The Cauchy Deformation Field in the Quaternion Representation

The Cauchy classical theory of elasticity is an elegant starting point to show the physical reality and the significance and beauty of quaternions. The Hamilton algebra Q allows recoupling the compression and twist that were separated in (16). Upon denoting $\sigma_{0}=\mathrm{div}u_{0}=\left(\sigma_{0},0,0,0\right)$ and $\hat{\phi }=\mathrm{rot}u_{\phi }=\left(0,\phi_{1},\phi_{2},\phi_{3}\right)$ as well $\hat{\phi }=\phi_{1}i+\phi_{2}j+\phi_{3}k$ we get

(17) $$\begin{array}{l} \frac{\partial^{2}\sigma_{0}}{\partial t^{2}}=3c^{2}\Delta \sigma_{0},\\ \frac{\partial^{2}\hat{\phi }}{\partial t^{2}}=c^{2}\Delta \hat{\phi }. \end{array}$$

Relation (15) takes the form

(18) $$e=1/2\dot{u}\circ \dot{u}+3/2c^{2}\sigma_{0}^{2}+1/2c^{2}\hat{\phi }\circ \hat{\phi }.$$

The decomposition $u=u_{0}+u_{\phi }$ in Equation (16) results in four equations in Equation (17) and implies the existence of the deformation field $\sigma =\sigma_{0}+\hat{\phi }$ that represents the twist and compression fields as a superposition of real (scalar compression $\sigma_{0}$) and imaginary (twist vector $\hat{\phi }$) field parts at each point

(19) $$\sigma =\sigma_{0}+\hat{\phi }\in Q\;\mathrm{and}\;\sigma^{*}=\sigma_{0}-\hat{\phi }\in Q,$$

where the following constraint holds

(20) $$\mathrm{div}\hat{\phi }=\mathrm{div}\mathrm{rot}u_{\phi }=0.$$

Adding equations in Equation (17) and from constraint (20), we get the quaternion form of the motion equation

(21) $$\frac{1}{c^{2}}\frac{\partial^{2}\sigma }{\partial t^{2}}-\Delta \sigma -2\Delta \sigma_{0}=0,\;\mathrm{where}\;\sigma =\sigma_{0}+\hat{\phi }.$$

Equation (21) must obey constraint (20) and require boundary conditions for a solution in the quaternion form. Since $\dot{u}\circ \dot{u}=\hat{\dot{u}}\circ \hat{\dot{u}}=-\hat{\dot{u}}\cdot \hat{\dot{u}}=\hat{\dot{u}}\cdot {\hat{\dot{u}}}^{*}$, where $\hat{\dot{u}}={\dot{u}}_{1}i+{\dot{u}}_{2}j+{\dot{u}}_{3}k$ and $\dot{u}=\left({\dot{u}}_{1},{\dot{u}}_{2},{\dot{u}}_{3}\right)$, the overall energy of the deformation field, the Formula (18) becomes in the quaternion form

(22) $$e=\frac{1}{2}\hat{\dot{u}}\cdot {\hat{\dot{u}}}^{*}+\frac{1}{2}c^{2}\sigma \cdot \sigma^{*}+c^{2}\sigma_{0}^{2}.$$

The energy is conserved, so relation (22) leads to the nonlocal boundary condition for Equation (21) [37].

**Remark** **3.** *The Cauchy theory of elastic continuum combined with the Helmholtz decomposition theorem and quaternion algebra results in second-order differential Equation (21) and constraint (20). It infers the transverse, longitudinal and multiple complex forms of waves. Equation (21) and the relation (22) satisfy the Eule–Lagrange differential equation, i.e., satisfy the fundamental equation of the calculus of variations.*

### 3.2. Quaternion Quantum Mechanics

In this section, we combine the Hamilton algebra of quaternions [50], the Cauchy classical mechanics in $R^{3}$ [41,42] with Planck–Kleinert crystal concept [34,35]. The fundamental new results, explicitly the ontology of Quaternion Quantum Mechanics, the appearance of imaginary numbers in Schrödinger equation and the family of waves in elastic continua will follow. 

The crystal hypothesis can be found in Maxwell paper published in 1856 “*A Dynamical Theory of the Electromagnetic Field”* [51] where Maxwell explicitly remarked on the ether:

“On our theory, it *(energy)* resides in the electromagnetic field, in the space surrounding the electrified and magnetic bodies, as well as in those bodies themselves, may be described according to a very probable hypothesis, as the motion and the strain of one and the same medium *(elastic ether)*.”

Less known, if not entirely forgotten, is the section on gravity where Maxwell wrote:

“…assumption, therefore, that gravitation arises from the action of the surrounding medium leads to the conclusion that every part of this medium possesses, when undisturbed, an enormous intrinsic energy As I am unable to understand in what way a medium can possess such properties, I cannot go any further in this direction in searching for the cause of gravitation.”

Maxwell was unsuccessful in formulating his theory in quaternion form. His idea of solid ether showing “enormous intrinsic energy” was unimaginable in the XIX-th century. 

In simple words, we regard quantum space as an analog to the Cauchy elastic solid. Already, upon adding Equation (17), we obtained quaternion form of the motion equation, Equation (21). In this section, we show that upon splitting Equation (21) into the system of the wave and Poisson type equations, the multiple non-linear forms of the wave equation follow. That is, the quaternion motion equation produces the family of the non-linear stationary and quasi-stationary waves. The properties of Planck–Kleinert crystal (Maxwell elastic ether) are presented in Table 1.

**Remark** **4.** *The Quaternion Quantum Mechanics follows from rigorous quaternion representation of the Cauchy linear theory of elasticity, Equations (20) and (21).*

### 3.3. Relativistic Stationary Waves in the Cauchy Continuum: the Quaternion Klein–Gordon Equation

To do so, we consider the wave showing the overall energy $E_{n}=\mathrm{const}$. Subsequently, Equation (21) can be written as a multisystem

(23) $$\left\{ \begin{array}{l} \frac{1}{c^{2}}\frac{\partial^{2}\sigma }{\partial t^{2}}-\left(n+1\right)\Delta \sigma +k_{n}^{2}\sigma \cdot \sigma^{*}=0,\\ \left(n-2\right)\Delta \sigma_{0}+n\Delta \hat{\phi }-k_{n}^{2}\sigma \cdot \sigma^{*}=0, \end{array}\right.\;\mathrm{for}\;n=0,1,2,\ldots$$

where $k_{n}=1/\lambda_{n}$ and $\lambda_{n}=f\left(E_{n}\right)$ denotes the wavelength. Note that by adding Equation in (23), the momentum balance is expressed again by a single partial differential Equation (21). The system (23) is a hyperbolic-elliptic quaternion representation of a the wave Equation (21) and have solutions of the form

(24) $$\sigma =\sigma_{0}+\hat{\phi }=\sigma_{0}+\phi_{1}i+\phi_{2}j+\phi_{3}k\in Q.$$

The second equation in (23) is the Poisson type equation, and it describes/defines the compression potential as a function of energy density. The analysis of the system (23) in a case when *n* = 0 shows that wave equation is the quaternion Klein–Gordon type equation [53] and Poisson equation

(25) $$\left\{ \begin{array}{l} \left(\frac{1}{c^{2}}\frac{\partial^{2}}{\partial t^{2}}-\Delta \right)\sigma +k_{n}^{2}\sigma^{*}\cdot \sigma =0,\\ 2\Delta \sigma_{0}=-k_{n}^{2}\sigma^{*}\cdot \sigma . \end{array}\right.$$

Consider a quasi-stationary wave in the Planck–Kleinert crystal, Table 1, i.e., the particle showing the constant overall energy (equivalent mass), $m=Ec^{-2}=const$, E denotes overall wave energy and $c=l_{P}/t_{P}$ [37]. Thus, Equation (25) can be written

(26) $$\left\{ \begin{array}{l} \left(\frac{\partial^{2}}{\partial t^{2}}-c^{2}\Delta \right)\sigma +\frac{8\pi m}{m_{P}t_{P}^{2}}\sigma^{*}\cdot \sigma =0,\\ 2c^{2}\Delta \sigma_{0}=-\frac{8\pi m}{m_{P}t_{P}^{2}}\sigma \cdot \sigma^{*}, \end{array}\right.$$

and the first equation above in the compacted form as

(27) $$\left(\frac{1}{c^{2}}\frac{\partial^{2}}{\partial t^{2}}-\Delta \right)\sigma +\frac{8\pi m}{\hbar t_{P}^{}}\sigma^{*}\cdot \sigma =0$$

or in the covariant notation

(28) $$\partial_{\mu }\partial^{\mu }\sigma +m\frac{8\pi m}{\hbar t_{P}^{}}\sigma^{*}\cdot \sigma =0,$$

where $\partial_{\mu }\partial^{\mu }=\frac{1}{c^{2}}\frac{\partial^{2}}{\partial t^{2}}-\Delta$ and $\hbar =m_{P}c^{2}t_{P}=1.0545727\times 10^{-34}\left[\mathrm{kg}\cdot m^{2}s^{-1}\right]$.

The Klein–Gordon equation fulfills the laws of special relativity, but contains two fundamental problems [54]. The first one is that it allows negative energies as a solution. As can be seen, the energy computed using Formulae (22) as well as Equation (28) is per definition always positive due to the constraint (20). Existence and properties of solutions to wave equations are studied for example in [55] and in the references therein.

The second equation in (26) is the Poisson equation and describes the irrotational, e.g., compression, potential in the deformation field. By introducing physical constants of the Planck–Kleinert crystal we get

(29) $$c^{2}\Delta \sigma_{0}=-4\pi m\frac{1}{m_{P}t_{P}^{2}}\sigma \cdot \sigma^{*}.$$

The above relation can be expressed as a function of the local mass density [37]: $\rho =\rho_{E}/c^{2}$, where $\rho_{E}=mc^{2}\sigma \cdot \sigma^{*}/l_{P}^{3}$

(30) $$c^{2}\Delta \sigma_{0}=-4\pi \rho \frac{l_{P}^{3}}{m_{P}t_{P}^{2}}=-4\pi \rho G,$$

where using data in Table 1, the gravitational constant equals: $G=l_{P}^{3}/\left(t_{P}^{2}m_{P}\right)=6.674082\times 10^{-11}$ $\left[m^{3}\cdot {\mathrm{kg}}^{-1}s^{-2}\right].$

**Remark** **5.** *The low deformation limit considered by us allows for the simplified assumption of the constant overall energy density as well as the constant transverse wave velocity. Consequently, the gravity in the simplified form of the Poisson equation follows. By considering the dependence of the energy density on deformation one can get the extended quaternion form of relation (29) or the geometrical theory of gravitation when the invariant velocity of a transverse wave is assumed.*

### 3.4. Non-Relativistic Waves in the Cauchy Continuum: the Quaternion Schrödinger Equation

The quasi-stationary wave means here that the wave can be treated as a particle in an arbitrary volume Ω. Such a wave has multiple properties, important here are:

1. the overall energy, $E=E^{0}+Q$, where $E^{0}$ and *Q* are the ground and excess energies, and the overall energy density, $\rho_{E}$;
2. the equivalent mass interrelated to the wave overall energy;
3. the wave mass center and its translation velocity υ [19].

In the following, the label kinetic will denote the wave translation in continuum: e.g., the wave velocity, υ. The category dynamic will denote the local movements within the elastic continuum itself, i.e., the local kinetic energy density, *k*, caused by the lattice local velocity, $\hat{\dot{u}}$. From relation (22), upon substituting ${\tilde{\sigma }}_{0}=\sqrt{3}\sigma_{0}^{}$, the overall wave energy can be expressed by the formula

(31) $$\begin{array}{ll} E=E^{0}+Q=\int_{\Omega }\rho_{E}dx & =c^{2}\int_{\Omega }\rho_{P}\left(\frac{1}{2c^{2}}\hat{\dot{u}}\cdot {\hat{\dot{u}}}^{*}+\frac{1}{2}\tilde{\sigma }\cdot {\tilde{\sigma }}^{*}\right)dx\\ & =c^{2}\int_{\Omega }\rho_{P}kdx+c^{2}\int_{\Omega }\rho_{P}sdx\\ & =K+S, \end{array}$$

where $\tilde{\sigma }={\tilde{\sigma }}_{0}+\hat{\sigma }=\sqrt{3}\sigma_{0}^{}+\hat{\sigma }.$

The overall mass of the particle, *m*, and the particle mass density, *ρ*, follow from (31),

(32) $$m=\int_{\Omega }\rho_{}dx=\int_{\Omega }\rho_{P}\left(\frac{1}{2c^{2}}\hat{\dot{u}}\cdot {\hat{\dot{u}}}^{*}+\frac{1}{2}\tilde{\sigma }\cdot {\tilde{\sigma }}^{*}\right)dx.$$

We conclude that the “the overall particle mass” follows from the relations (31) and (32): $m=E/c^{2}$. The *mass* is ontic in that sense that the particle mass is identified with the overall dynamic energy of the wave in elastic quasi-continuum of the Planck particles, see also [56]. Note that in general when *Q* > 0, *m* differs from the mass at the ground state. In simple words, when a Planck particle in the ideal crystal due to an arbitrary cyclic process has the overall energy $E_{}^{P}$, then the ratio $m=E_{}^{P}/c^{2}$ we call the mass of the “particle localized on” this single Planck particle.

The waves in Ω may differ in shape and other essentials, the overall particle energy, relation (31), reveals the wave nature. The considered here quasi-stationary wave has to satisfy the relation (31) and at every position, the energy density is a sum of the dynamic, *k*, and strain, *s*, energy terms: $\rho_{E}=c^{2}\rho_{P}\left(k+s\right)=0.4Y\left(k+s\right)$.

We start with an elementary situation when the velocity of the wave mass center υ is low and constant, υ≪c. By using the extremum principle, namely the action concept, one can quantify the elementary properties of such waves. At every position in Ω:

1. the existence assumption of the quasi-stationary wave implies an equal duration of the periodic cycles in the whole volume occupied by the wave, Δt=const. Consequently implies, that the *s*- and *k*-actions are equal
  (33) $$\int_{t}^{t+\Delta t}s\left(\tau ,x\right)d\tau =\int_{t}^{t+\Delta t}k\left(\tau ,x\right)d\tau =\gamma \left(x\right)\Delta t;$$
2. the sum of the overall strain, *S*, and the kinetic energy, *K*, in relation (31) equals the overall wave energy $E=E^{0}+Q$, and is time-invariant;
3. spans of the strain and the kinetic energy terms are equal,
  $$\left[0,\max\left\{k\left(t,x\right)\right\}\right]=\left[0,\max\left\{s\left(t,x\right)\right\}\right]=\left[0,\rho_{E}\left(t,x\right)/\left(c^{2}\rho_{P}\right)\right]=\left[0,\rho_{E}\left(t,x\right)/\left(0.4Y\right)\right].$$

The relation (33) is valid for the whole Ω so

$$\int_{t}^{t+\Delta t}\int_{\Omega }s\left(\tau ,x\right)dxd\tau =\int_{t}^{t+\Delta t}\int_{\Omega }k\left(\tau ,x\right)dxd\tau =\gamma_{\Omega }\Delta t$$

and also for an arbitrary number of cycles: t=nΔt. Thus, from assumptions 2 and 3 above and the relation (31), it follows that both actions in Ω can be approximated by the discrete formula

(34) $$\gamma_{\Omega }n\Delta t=\int_{0}^{n\Delta t}\int_{\Omega }sdxd\tau =\int_{0}^{n\Delta t}\int_{\Omega }kdxd\tau .$$

Taking into account that we consider time evolution in a case when t≫Δt, the continuous expressions for both actions follow

(35) $$\gamma_{\Omega }t=\int_{0}^{t}\int_{\Omega }sdxd\tau =\int_{0}^{t}\int_{\Omega }kdxd\tau .$$

Taking time derivative of the relations in (35) we get:

(36) $$\int_{\Omega }sdx=\int_{\Omega }kdx\;\mathrm{for}\;t\gg \Delta t.$$

Both terms, *s* and *k*, in (36) oscillate and depend on the time and position. It will be useful to normalize the displacement term *s* in (31) with respect to the overall particle mass, relation (32). Note that because we restrict our analysis to the low velocities υ≪c, the translation energy has a minor impact on the overall wave mass and $m≅m_{0}$. From the formulas (31), (32) and (36), the normalized strain energy density, *s*, equals

(37) $$\int_{\Omega }\frac{\rho_{P}}{m}\tilde{\sigma }\cdot {\tilde{\sigma }}^{*}dx=\int_{\Omega }\psi \cdot \psi^{*}dx=1,\;\mathrm{where}\;\psi =\sqrt{\frac{\rho_{P}}{m}}\tilde{\sigma }.$$

Consequently from (31), (36) and (37)

(38) $$0=\int_{\Omega }\left(\rho_{P}c^{2}\tilde{\sigma }\cdot {\tilde{\sigma }}^{*}-E\psi \cdot \psi^{*}\right)dx$$

and also

(39) $$0=\int_{\Omega }\left[\rho_{P}\hat{\dot{u}}\cdot {\hat{\dot{u}}}^{*}-E\psi \cdot \psi^{*}\right]dx.$$

Note that, the relations (31), (32) and (38) imply the relation between the overall energy of the wave, and equivalently the overall wave mass $E=mc^{2},$ and the probability density because the relation (37) is satisfied. Obviously, the both terms, $\psi =\sqrt{\frac{\rho_{P}}{m}}\tilde{\sigma }$ and $\psi \cdot \psi^{*},$ vary in time.

**Remark** **6.** *The relation (38) links up frequency of the wave with its overall energy:*

*(1)*  *the excess, Q, and ground, $E^{0}$, energies are entangled in (38) and can’t be separated;*
*(2)*  *the overall wave energy can be increased by translation term, accordingly also all displacements and velocities are affected;*
*(3)*  *the wave periodicity implies that by solving the relation (39), one should expect only the discrete values if excess energy Q;*
*(4)*  *when the wave overall energy equals its ground energy, Q = 0, then relation (38) results in*
  (40) $$0=\int_{\Omega }\left(\rho_{P}c^{2}{\tilde{\sigma }}^{0}\cdot {\left({\tilde{\sigma }}^{0}\right)}^{*}-E^{0}\psi \cdot \psi^{*}\right)dx,$$
  *where ${\tilde{\sigma }}^{0}$ denotes the displacement in the wave at the ground energy $E^{0}$.*

### 3.5. Waves in the Time-Invariant Potential Field

Let us consider now the evolution of the wave as in the relation (31) in the time-invariant potential field, e.g., in the field generated by other particles. The overall energy is now a sum of the ground and excess energy

(41) $$E=E^{0}+Q=\int_{\Omega }\left(\frac{1}{2}\rho_{P}\hat{\dot{u}}\cdot {\hat{\dot{u}}}^{*}+\frac{1}{2}\rho_{P}c^{2}\tilde{\sigma }\cdot {\tilde{\sigma }}^{*}+V\left(x\right)\psi \cdot \psi^{*}\right)dx,$$

where $\sqrt{\tilde{\sigma }\cdot {\tilde{\sigma }}^{*}}=\left\|{\tilde{\sigma }}^{0}+{\tilde{\sigma }}_{Q}\right\|=\left\|\tilde{\sigma }\right\|$ and $\tilde{\sigma }={\tilde{\sigma }}^{0}+{\tilde{\sigma }}_{Q}$.

We consider low excess energy, υ≪c. Consequently: (1) the impact of *Q* on the overall particle mass is marginal, $m≅m_{0}$, and (2) the displacement $\tilde{\sigma }$ in (41) can be normalized using the formula (37). Thus relation (41) becomes

(42) $$\begin{array}{ll} E=E^{0}+Q & =\int_{\Omega }\left(\frac{1}{2}\rho_{P}\hat{\dot{u}}\cdot {\hat{\dot{u}}}^{*}+\frac{1}{2}mc^{2}\psi \cdot \psi^{*}+V\left(x\right)\psi \cdot \psi^{*}\right)dx\\ & =\frac{1}{2}mc^{2}+\int_{\Omega }\left(\frac{1}{2}\rho_{P}\hat{\dot{u}}\cdot {\hat{\dot{u}}}^{*}+V\left(x\right)\psi \cdot \psi^{*}\right)dx. \end{array}$$

Both, the $E^{0}$ and *m* are constant, thus it is enough to minimize the relation

(43) $$Q=\int_{\Omega }\left(\frac{1}{2}\rho_{P}\hat{\dot{u}}\cdot {\hat{\dot{u}}}^{*}+V\left(x\right)\psi \cdot \psi^{*}\right)dx.$$

The above relation contains the unknown kinetic velocity due to the potential V(x). The Cauchy–Riemann operator of the deformation, $D\tilde{\sigma }$, can be understood, by means of the relations (11) and (12), as an analogy of the gradient in $R^{3}$. In classical dynamics, the potential gradient results in acceleration. For the quaternion representation of the deformation field, it is reasonable to guess that the local momentum in the crystal (i.e., the Planck particle momentum), $\hat{p}=m_{p}\hat{\dot{u}}$, is related to the Cauchy–Riemann operator of the quaternion displacement, $D\tilde{\sigma }.$ Namely, the local lattice velocity is proportional to (1) the force that is the normalized Cauchy–Riemann derivative of the local displacement, $l_{P}D\tilde{\sigma }$, and (2) the transverse wave velocity c. Accordingly

(44) $$\hat{p}=-m_{P}cl_{P}D\tilde{\sigma }=-\hbar D\tilde{\sigma },$$

where we introduced constants $\hbar =m_{P}c^{2}t_{P}$ and $t_{P}=l_{P}/c$ i.e., is the time that transverse wave travels at the lattice distance: $l_{P}=ct_{P}$.

The momentum balance requires

(45) $$\hat{\dot{u}}=\frac{\hat{p}}{m}=-\frac{\hbar }{m}D\tilde{\sigma }.$$

By introducing (45), the relation (43) becomes

(46) $$Q=\int_{\Omega }\left(\rho_{P}\frac{\hbar^{2}}{2m^{2}}\left(D\tilde{\sigma }\right)\cdot {\left(D\tilde{\sigma }\right)}^{*}+V\left(x\right)\psi \cdot \psi^{*}\right)dx.$$

Normalization using (37) results in the functional

(47) $$Q[\psi ]=\int_{\Omega }\left(\frac{\hbar^{2}}{2m}\left(D\psi \right)\cdot {\left(D\psi \right)}^{*}+V\left(x\right)\psi \cdot \psi^{*}\right)dx.$$

There are numerous methods for solving the above problem, e.g., the path integrals, the Hamilton Jacobi equation, etc. We minimize the functional Q[ψ], that is the integral above, with respect to a quaternion function such that ψ satisfies the normalization introduced in the relation (37). We look for a differential equation that has to be satisfied by the ψ function to extremize (here minimize) the energies allowed by (47). Subsequently, we will show that the extremum problem leads to the quaternion analog of the time-independent Schrödinger equation. 

Given the functional (47), we seek for the conditional extreme and use the Lagrange coefficients method combined with a procedure presented in Appendix A. Then, there exists a multiplayer λ≠0 such that ψ minimalizes the functional

(48) $$\tilde{Q}\left[\psi \right]=\int_{\Omega }\left[\frac{\hbar^{2}}{2m}\left(D\psi \right)\cdot {\left(D\psi \right)}^{*}+V\left(x\right)\psi \cdot \psi^{*}+\lambda \left(\frac{1}{\left|\Omega \right|}-\psi \cdot \psi^{*}\right)\right]dx.$$

It follows from Appendix A that ψ satisfies the differential equation

(49) $$-\frac{\hbar^{2}}{2m}\Delta \psi +V\left(x\right)\psi =\lambda \psi ,$$

where a constant factor on the right-hand side can be considered as extra energy of the particle in the presence of the field *V* = *V*(*x*). 

For E=λ, Equation (49) is clearly the time-independent Schrödinger equation satisfied by the particle in the ground state of the energy E,

(50) $$-\frac{\hbar^{2}}{2m}\Delta \psi +V\left(x\right)\psi =E\psi .$$

It has to be satisfied together with the condition

(51) $$\mathrm{div}\hat{\psi }=0\;\mathrm{where}\;\psi =\psi_{0}+\hat{\psi }.$$

Upon using the NIST data [52] of the Planck’s natural units $m_{P},\;l_{P},\;t_{p}$ and the light velocity *c*, the constant ℏ introduced in relation (44) equals the Planck constant, $\hbar =6.626069311\times 10^{-34}$.

The particle (wave) mass center. The meaning assigned to “space-localized” is used in the sense given by the Bodurov definition [57]:

A singularity-free multi-component function $\sigma =\left(\sigma_{0},\phi_{1},\phi_{2},\phi_{3}\right)\in Q$ of the space $x=\left(x_{1},x_{2},x_{3}\right)$ and time *t* variables will be called space-localized if ‖σ(t,x)‖→0 sufficiently fast when ‖x‖→∞, so that its Hermitean norm, see Equation (9)

(52) $$\langle \sigma ,\sigma^{*}\rangle =\int_{\Omega }\left(\sigma_{0}^{2}+\sum_{l=1}^{3}\phi_{l}\cdot \phi_{l}^{*}\right)dx=\int_{\Omega }\sigma \cdot \sigma^{*}dx<\infty ,$$

remains finite for all time.

### 3.6. Quaternion Time Dependent Schrödinger Equation

By analogy to the complex time-dependent Schrödinger equation $i\hbar \frac{\partial \Psi }{\partial t}=-\frac{\hbar^{2}}{2m}\Delta \Psi +V\left(x\right)\Psi$, we propose the quaternion form

(53) $$\frac{1}{3}\left(i+j+k\right)\hbar \frac{\partial \Psi }{\partial t}=-\frac{\hbar^{2}}{2m}\Delta \Psi +V\left(x\right)\Psi .$$

We will show now that by the substitution $\Psi \left(t,x\right)=e^{-\left(i+j+k\right)\frac{E}{\hbar }t}\psi \left(x\right)$, the Equation (53) leads to the time-independent Schrödinger Equation (50). Note that by the trigonometric Formula (13), we have

(54) $$\Psi \left(t,x\right)=\left[\cos\left(\sqrt{3}\frac{E}{\hbar }t\right)-\frac{1}{\sqrt{3}}\left(i+j+k\right)\sin\left(\sqrt{3}\frac{E}{\hbar }t\right)\right]\psi \left(x\right),$$

(55) $$\begin{array}{ll} \frac{\partial \Psi }{\partial t}\left(t,x\right) & =\left[-\sqrt{3}\frac{E}{\hbar }\sin\left(\sqrt{3}\frac{E}{\hbar }t\right)-\left(i+j+k\right)\frac{E}{\hbar }\cos\left(\sqrt{3}\frac{E}{\hbar }t\right)\right]\psi \left(x\right)\\ & =-\left(i+j+k\right)\frac{E}{\hbar }\left[\cos\left(\sqrt{3}\frac{E}{\hbar }t\right)-\frac{1}{\sqrt{3}}\left(i+j+k\right)\sin\left(\sqrt{3}\frac{E}{\hbar }t\right)\right]\psi \left(x\right)\\ & =-\left(i+j+k\right)\frac{E}{\hbar }\cdot e^{-\left(i+j+k\right)\frac{E}{\hbar }t}\psi \left(x\right). \end{array}$$

Obviously

(56) $$\Delta \Psi \left(t,x\right)=e^{-\left(i+j+k\right)\frac{E}{\hbar }t}\Delta \psi \left(x\right).$$

Hence it is immediately seen that Equation (53) implies Equation (50). Consider the special case $\Psi_{1}=\Psi_{2}=\Psi_{3}$ and put $\tilde{\Psi }:=\Psi_{1}=\Psi_{2}=\Psi_{3}$. It follows from elementary calculations that $\Psi :=\Psi_{0}+\frac{i+j+k}{\sqrt{3}}\tilde{\Psi }$ solves the quaternion Schrödinger Equation (53) if and only if $\Psi :=\Psi_{0}+i\tilde{\Psi }$ solves the complex Schrödinger equation

(57) $$\frac{1}{\sqrt{3}}i\hbar \frac{\partial \Psi }{\partial t}=-\frac{\hbar^{2}}{2m}\Delta \Psi +V\left(x\right)\Psi .$$

**Remark** **7.** *The quaternion form of the time-dependent Schrödinger Equation (53) was given. In the very special diagonal case it was demonstrated that the quaternion and complex time dependent Schrödinger equations, (53) and (57), are equivalent in same sense. Moreover, it was shown that by suitable natural substitution, the time-dependent Schrödinger Equation (53) implies the quaternion stationary Schrödinger Equation (50).*

## 4. Summary

We presented the foundation of Quaternion Quantum Mechanics based on the Cauchy model of the elastic continuum. Cauchy model of an ideal elastic solid with the Helmholtz decomposition theorem and the quaternion algebra Q generates the transverse, longitudinal and multiple forms of waves. The quaternionic analog of vector formulation of the Cauchy model elucidates the coupling between the irrotational and solenoidal displacements in the deformation field (compression and torsion) and allows for a physical interpretation of the wave mechanics. The wave, i.e., the collective movement of the constituents forming the elastic Navier–Cauchy continuum, is considered equivalent to the particle. By combining the quaternion representation of the Cauchy model with the Planck–Kleinert crystal concept we presented the self-consistent formulation of the wave phenomena where the quantum space is regarded as an analog to the elastic solid. The presented Quaternion Quantum Mechanics follows from rigorous quaternion representation of the Cauchy linear theory of elasticity:

1. The quaternion form of the time-dependent Schrödinger Equation (53) was obtained and the special case when it solves complex Schrödinger equation was demonstrated. Thus, the origin of complex numbers in QM was explained.
2. The Klein–Gordon and Poisson equations were derived from assumptions that are independent of the postulates of quantum mechanics and prove the origin of the wave function. The problem of the indefinite probability of the density, present in the classical Klein–Gordon equation, is ruled out in its quaternion form.
3. All presented family of the quaternion wave functions is ontic, directly represent a state of elastic continuum showing properties of the Planck–Kleinert crystal.

The method used by us allows the self-consistent interpretation of the wave phenomena and yields the non-relativistic gravity field. It is obvious that it can be generalized and extended upon neglecting the assumptions of the small deformation limit that implies the constant density and the constant transport properties within the deformation field. The model can be falsified, see K. Popper [58]. Namely, the following hypotheses require formal verification:

1. Experimental verification to reveal superdeterminism, e.g., using the method proposed by Hossenfelder [59].
2. The experimental verification of the particle mass center, Equation (52).
3. The avoiding of the assumption of the small deformation limit implies the varying transverse wave velocity. The dependence of the energy density on deformation will result in:
  - the extended quaternion form of relation (29) and
  - the geometrical theory of gravitation when an invariant velocity of the transverse wave is avoided.
4. A reexamination of Schrödinger’s charge density hypothesis. Namely, the Noether theorem can be used to decrease the order of the quaternion form of the Schrödinger equation [60].
5. The time-dependent Schrödinger requires the rigorous proof of the quaternion form of the Hamilton–Jacobi equation.

What is more, the results demonstrate that quaternions are much more comfortable than vectors, have huge advantages in the calculation of twist (and rotations) and can be regarded as the most concise representation of physical reality. Not only at the Planck scale, not only helpful and convenient, but quaternions also allow us to understand the processes in continua.

## Figures and Tables

**Table 1:** The physical constants of the Planck–Kleinert crystal (fcc ideal isotropic crystal).

Label Used in This Work	Planck Constants	Symbol for Unit	Value	SI Unit	Reference
Transverse wave velocity	Light velocity in vacuum	*c*	$2.99792458\times 10^{8}$	m∙s^−1^	[52]
Lattice parameter	Planck length	$l_{P}$	1.616229(38) × 10^−35^	m	[52]
Poisson ratio		ν	0.25	-	[52]
Mass of the Planck particle	Planck mass	$m_{P}$	2.176470(51) × 10^−8^	kg	[52]
Planck–Kleinert crystal density		$\rho_{P}$	2.062072 × 10^97^	kg∙m^−3^	[52]
Duration of the internal process	Planck time	$t_{P}$	5.39116(13) × 10^−44^	s^−1^	[52]
Young modulus	Intrinsic energy density	*Y*	4.6332447 × 10^114^	kg∙m^−1^s^−2^	$c=\sqrt{0.4Y/\rho_{P}}$

## Appendix A

Let $\Omega \subset R^{3}$ be an open bounded set with the smooth boundary ∂Ω. Moreover, let $\alpha ,\beta :\bar{\Omega }\to R$ be given sufficiently regular functions, in a special case they can be constant. Define the real functional

(A1) $$F\left[\psi \right]=\int_{\Omega }\left[\alpha \left(x\right)\psi \cdot \psi^{*}+\beta \left(x\right)\left(D\psi \right)\cdot {\left(D\psi \right)}^{*}\right]dx$$

acting on the set $S=\left\{\psi :\bar{\Omega }\to H\;\mathrm{of}\;\mathrm{the}\;C^{2}\;\mathrm{class}\right\}\cap \left\{\psi =g\;\mathrm{on}\;\partial \Omega \right\}$, where *g* is a given function. The functional *F* can be written in the form

$$\begin{array}{ll} F\left[\psi \right] & =\int_{\Omega }\left[\alpha \left(x\right){\left\|\psi \right\|}^{2}+\beta \left(x\right)\left({\left\|\nabla \psi_{0}\right\|}^{2}+{\left\|\mathrm{rot}\hat{\psi }\right\|}^{2}\right)+2\beta \left(x\right)\mathrm{rot}\hat{\psi }\circ \nabla \psi_{0}+2\beta \left(x\right){\left(\mathrm{div}\hat{\psi }\right)}^{2}\right]dx\\ & =\int_{\Omega }\{\alpha \left(x\right)\left(\psi_{0}^{2}+\psi_{1}^{2}+\psi_{2}^{2}+\psi_{3}^{2}\right)+\beta \left(x\right)\left[{\left(\frac{\partial \psi_{0}}{\partial x_{1}}\right)}^{2}+{\left(\frac{\partial \psi_{0}}{\partial x_{2}}\right)}^{2}+{\left(\frac{\partial \psi_{0}}{\partial x_{3}}\right)}^{2}\right]\\ & \;\;+\beta \left(x\right)\left[{\left(\frac{\partial \psi_{2}}{\partial x_{3}}-\frac{\partial \psi_{3}}{\partial x_{2}}\right)}^{2}+{\left(\frac{\partial \psi_{3}}{\partial x_{1}}-\frac{\partial \psi_{1}}{\partial x_{3}}\right)}^{2}+{\left(\frac{\partial \psi_{1}}{\partial x_{2}}-\frac{\partial \psi_{2}}{\partial x_{1}}\right)}^{2}\right]\\ & \;\;+2\beta \left(x\right)\left[\left(\frac{\partial \psi_{2}}{\partial x_{3}}-\frac{\partial \psi_{3}}{\partial x_{2}}\right)\frac{\partial \psi_{0}}{\partial x_{1}}+\left(\frac{\partial \psi_{3}}{\partial x_{1}}-\frac{\partial \psi_{1}}{\partial x_{3}}\right)\frac{\partial \psi_{0}}{\partial x_{2}}+\left(\frac{\partial \psi_{1}}{\partial x_{2}}-\frac{\partial \psi_{2}}{\partial x_{1}}\right)\frac{\partial \psi_{0}}{\partial x_{3}}\right]\\ & \;\;+\beta \left(x\right){\left(\frac{\partial \psi_{1}}{\partial x_{1}}+\frac{\partial \psi_{2}}{\partial x_{2}}+\frac{\partial \psi_{3}}{\partial x_{3}}\right)}^{2}\}dx. \end{array}$$

Suppose that ψ minimizes *F* on *S*. We will show that ψ solves some differential equation. Let $\varphi :\bar{\Omega }\to H$ be any smooth function and φ=0 on ∂Ω, i.e., $\varphi \in C_{0}^{\infty }\left(\bar{\Omega },H\right)$. Define a real function

(A2) f:R→R, f(τ)=F[ψ+τφ]

Obviously $f^{\prime }\left(0\right)=0$, because ψ+τφ∈S and f has a minimum in τ=0.

We make the calculations:

$$\begin{array}{ll} f\left(\tau \right)= & \int_{\Omega }\{\alpha \left(x\right)\left[{\left(\psi_{0}^{}+\tau \varphi_{0}\right)}^{2}+{\left(\psi_{1}^{}+\tau \varphi_{1}\right)}^{2}+{\left(\psi_{2}^{}+\tau \varphi_{2}\right)}^{2}+{\left(\psi_{3}^{}+\tau \varphi_{3}\right)}^{2}\right]\\ & +\beta \left(x\right)\left[{\left(\frac{\partial \psi_{0}}{\partial x_{1}}+\tau \frac{\partial \varphi_{0}}{\partial x_{1}}\right)}^{2}+{\left(\frac{\partial \psi_{0}}{\partial x_{2}}+\tau \frac{\partial \varphi_{0}}{\partial x_{2}}\right)}^{2}+{\left(\frac{\partial \psi_{0}}{\partial x_{3}}+\tau \frac{\partial \varphi_{0}}{\partial x_{3}}\right)}^{2}\right]\\ & +\beta \left(x\right)[{\left(\frac{\partial \psi_{2}}{\partial x_{3}}+\tau \frac{\partial \varphi_{2}}{\partial x_{3}}-\frac{\partial \psi_{3}}{\partial x_{2}}-\tau \frac{\partial \varphi_{3}}{\partial x_{2}}\right)}^{2}\\ & \;+{\left(\frac{\partial \psi_{3}}{\partial x_{1}}+\tau \frac{\partial \varphi_{3}}{\partial x_{1}}-\frac{\partial \psi_{1}}{\partial x_{3}}-\tau \frac{\partial \varphi_{1}}{\partial x_{3}}\right)}^{2}+{\left(\frac{\partial \psi_{1}}{\partial x_{2}}+\tau \frac{\partial \varphi_{1}}{\partial x_{2}}-\frac{\partial \psi_{2}}{\partial x_{1}}-\tau \frac{\partial \varphi_{2}}{\partial x_{1}}\right)}^{2}]\\ & +2\beta \left(x\right)[\left(\frac{\partial \psi_{2}}{\partial x_{3}}+\tau \frac{\partial \varphi_{2}}{\partial x_{3}}-\frac{\partial \psi_{3}}{\partial x_{2}}-\tau \frac{\partial \varphi_{3}}{\partial x_{2}}\right)\left(\frac{\partial \psi_{0}}{\partial x_{1}}+\tau \frac{\partial \varphi_{0}}{\partial x_{1}}\right)\\ & \;+\left(\frac{\partial \psi_{3}}{\partial x_{1}}+\tau \frac{\partial \varphi_{3}}{\partial x_{1}}-\frac{\partial \psi_{1}}{\partial x_{3}}-\tau \frac{\partial \varphi_{1}}{\partial x_{3}}\right)\left(\frac{\partial \psi_{0}}{\partial x_{2}}+\tau \frac{\partial \varphi_{0}}{\partial x_{2}}\right)\\ & \;+\left(\frac{\partial \psi_{1}}{\partial x_{2}}+\tau \frac{\partial \varphi_{1}}{\partial x_{2}}-\frac{\partial \psi_{2}}{\partial x_{1}}-\tau \frac{\partial \varphi_{2}}{\partial x_{1}}\right)\left(\frac{\partial \psi_{0}}{\partial x_{3}}+\tau \frac{\partial \varphi_{0}}{\partial x_{3}}\right)]\\ & +\beta \left(x\right){\left[\frac{\partial \psi_{1}}{\partial x_{1}}+\tau \frac{\partial \varphi_{1}}{\partial x_{1}}+\frac{\partial \psi_{2}}{\partial x_{2}}+\tau \frac{\partial \varphi_{2}}{\partial x_{2}}+\frac{\partial \psi_{3}}{\partial x_{3}}+\tau \frac{\partial \varphi_{3}}{\partial x_{3}}\right]}^{2}\}dx, \end{array}$$

$$\begin{array}{ll} 0=f^{\prime }\left(0\right)= & 2\int_{\Omega }\{\alpha \left(x\right)\left(\psi_{0}^{}\varphi_{0}+\psi_{1}^{}\varphi_{1}+\psi_{2}^{}\varphi_{2}+\psi_{3}^{}\varphi_{3}\right)+\beta \left(x\right)\left(\frac{\partial \psi_{0}}{\partial x_{1}}\frac{\partial \varphi_{0}}{\partial x_{1}}+\frac{\partial \psi_{0}}{\partial x_{2}}\frac{\partial \varphi_{0}}{\partial x_{2}}+\frac{\partial \psi_{0}}{\partial x_{3}}\frac{\partial \varphi_{0}}{\partial x_{3}}\right)\\ & +\beta \left(x\right)[\left(\frac{\partial \psi_{2}}{\partial x_{3}}-\frac{\partial \psi_{3}}{\partial x_{2}}\right)\left(\frac{\partial \varphi_{2}}{\partial x_{3}}-\frac{\partial \varphi_{3}}{\partial x_{2}}\right)+\left(\frac{\partial \psi_{3}}{\partial x_{1}}-\frac{\partial \psi_{1}}{\partial x_{3}}\right)\left(\frac{\partial \varphi_{3}}{\partial x_{1}}-\frac{\partial \varphi_{1}}{\partial x_{3}}\right)\\ & \;+\left(\frac{\partial \psi_{1}}{\partial x_{2}}-\frac{\partial \psi_{2}}{\partial x_{1}}\right)\left(\frac{\partial \varphi_{1}}{\partial x_{2}}-\frac{\partial \varphi_{2}}{\partial x_{1}}\right)]\\ & +\beta \left(x\right)[\left(\frac{\partial \varphi_{2}}{\partial x_{3}}-\frac{\partial \varphi_{3}}{\partial x_{2}}\right)\frac{\partial \psi_{0}}{\partial x_{1}}+\left(\frac{\partial \psi_{2}}{\partial x_{3}}-\frac{\partial \psi_{3}}{\partial x_{2}}\right)\frac{\partial \varphi_{0}}{\partial x_{1}}+\left(\frac{\partial \varphi_{3}}{\partial x_{1}}-\frac{\partial \varphi_{1}}{\partial x_{3}}\right)\frac{\partial \psi_{0}}{\partial x_{2}}\\ & \;+\left(\frac{\partial \psi_{3}}{\partial x_{1}}-\frac{\partial \psi_{1}}{\partial x_{3}}\right)\frac{\partial \varphi_{0}}{\partial x_{2}}+\left(\frac{\partial \varphi_{1}}{\partial x_{2}}-\frac{\partial \varphi_{2}}{\partial x_{1}}\right)\frac{\partial \psi_{0}}{\partial x_{3}}+\left(\frac{\partial \psi_{1}}{\partial x_{2}}-\frac{\partial \psi_{2}}{\partial x_{1}}\right)\frac{\partial \varphi_{0}}{\partial x_{3}}]\\ & +\beta \left(x\right)\left(\frac{\partial \psi_{1}}{\partial x_{1}}+\frac{\partial \psi_{2}}{\partial x_{2}}+\frac{\partial \psi_{3}}{\partial x_{3}}\right)\left(\frac{\partial \varphi_{1}}{\partial x_{1}}+\frac{\partial \varphi_{2}}{\partial x_{2}}+\frac{\partial \varphi_{3}}{\partial x_{3}}\right)\}dx. \end{array}$$

After differentiation by parts we have

$$\begin{array}{l} \int_{\Omega }\{\alpha \left(x\right)\left(\psi_{0}^{}\varphi_{0}+\psi_{1}^{}\varphi_{1}+\psi_{2}^{}\varphi_{2}+\psi_{3}^{}\varphi_{3}\right)-\beta \left(x\right)\left(\Delta \psi_{0}\right)\varphi_{0}+\beta \left(x\right)\{\left[\frac{\partial }{\partial x_{3}}\left(\frac{\partial \psi_{3}}{\partial x_{1}}-\frac{\partial \psi_{1}}{\partial x_{3}}\right)-\frac{\partial }{\partial x_{2}}\left(\frac{\partial \psi_{1}}{\partial x_{2}}-\frac{\partial \psi_{2}}{\partial x_{1}}\right)\right]\varphi_{1}\\ \;+\left[\frac{\partial }{\partial x_{1}}\left(\frac{\partial \psi_{1}}{\partial x_{2}}-\frac{\partial \psi_{2}}{\partial x_{1}}\right)-\frac{\partial }{\partial x_{3}}\left(\frac{\partial \psi_{2}}{\partial x_{3}}-\frac{\partial \psi_{3}}{\partial x_{2}}\right)\right]\varphi_{2}+\left[\frac{\partial }{\partial x_{2}}\left(\frac{\partial \psi_{2}}{\partial x_{3}}-\frac{\partial \psi_{3}}{\partial x_{2}}\right)-\frac{\partial }{\partial x_{1}}\left(\frac{\partial \psi_{3}}{\partial x_{1}}-\frac{\partial \psi_{1}}{\partial x_{3}}\right)\right]\varphi_{3}\}\\ \;+\beta \left(x\right)\{[-\frac{\partial }{\partial x_{1}}\left(\frac{\partial \psi_{2}}{\partial x_{3}}-\frac{\partial \psi_{3}}{\partial x_{2}}\right)-\frac{\partial }{\partial x_{2}}\left(\frac{\partial \psi_{3}}{\partial x_{1}}-\frac{\partial \psi_{1}}{\partial x_{3}}\right)-\frac{\partial }{\partial x_{3}}\left(\frac{\partial \psi_{1}}{\partial x_{2}}-\frac{\partial \psi_{2}}{\partial x_{1}}\right)]\varphi_{0}\\ \;+[\left(\frac{\partial^{2}\psi_{0}}{\partial x_{3}\partial x_{2}}-\frac{\partial^{2}\psi_{0}}{\partial x_{2}\partial x_{3}}\right)\varphi_{1}+\left(\frac{\partial^{2}\psi_{0}}{\partial x_{1}\partial x_{3}}-\frac{\partial^{2}\psi_{0}}{\partial x_{3}\partial x_{1}}\right)\varphi_{2}+\left(\frac{\partial^{2}\psi_{0}}{\partial x_{2}\partial x_{1}}-\frac{\partial^{2}\psi_{0}}{\partial x_{1}\partial x_{2}}\right)\varphi_{3}]\}\\ \;+\beta \left(x\right)\left[-\left(\frac{\partial }{\partial x_{1}}\mathrm{div}\hat{\psi }\right)\varphi_{1}-\left(\frac{\partial }{\partial x_{2}}\mathrm{div}\hat{\psi }\right)\varphi_{2}-\left(\frac{\partial }{\partial x_{3}}\mathrm{div}\hat{\psi }\right)\varphi_{3}\right]\}dx=0. \end{array}$$

The last equation takes the form

$$\begin{array}{l} \int_{\Omega }\{\alpha \left(x\right)\left(\psi_{0}^{}\varphi_{0}+\psi_{1}^{}\varphi_{1}+\psi_{2}^{}\varphi_{2}+\psi_{3}^{}\varphi_{3}\right)-\beta \left(x\right){\left(\Delta \psi_{0}^{}\right)}_{}^{}\varphi_{0}\\ \;+\beta \left(x\right)(\left[\frac{\partial }{\partial x_{3}}\left(\frac{\partial \psi_{3}}{\partial x_{1}}-\frac{\partial \psi_{1}}{\partial x_{3}}\right)-\frac{\partial }{\partial x_{2}}\left(\frac{\partial \psi_{1}}{\partial x_{2}}-\frac{\partial \psi_{2}}{\partial x_{1}}\right)-\frac{\partial }{\partial x_{1}}\mathrm{div}\hat{\psi }\right]\varphi_{1}\\ \;+\left[\frac{\partial }{\partial x_{1}}\left(\frac{\partial \psi_{1}}{\partial x_{2}}-\frac{\partial \psi_{2}}{\partial x_{1}}\right)-\frac{\partial }{\partial x_{3}}\left(\frac{\partial \psi_{2}}{\partial x_{3}}-\frac{\partial \psi_{3}}{\partial x_{2}}\right)-\frac{\partial }{\partial x_{2}}\mathrm{div}\hat{\psi }\right]\varphi_{2}\\ \;+\left[\frac{\partial }{\partial x_{2}}\left(\frac{\partial \psi_{2}}{\partial x_{3}}-\frac{\partial \psi_{3}}{\partial x_{2}}\right)-\frac{\partial }{\partial x_{1}}\left(\frac{\partial \psi_{3}}{\partial x_{1}}-\frac{\partial \psi_{1}}{\partial x_{3}}\right)-\frac{\partial }{\partial x_{3}}\mathrm{div}\hat{\psi }\right]\varphi_{3})\}dx=0. \end{array}$$

Equivalently we can write

(A3) $$\begin{array}{l} \int_{\Omega }\{{\left[\alpha \left(x\right)\psi_{0}-\beta \left(x\right)\Delta \psi_{0}\right]}^{}\varphi_{0}+\left[\alpha \left(x\right)\psi_{1}-\beta \left(x\right)\Delta \psi_{1}\right]\varphi_{1}\\ \;+\left[\alpha \left(x\right)\psi_{2}-\beta \left(x\right)\Delta \psi_{2}\right]\varphi_{2}+{\left[\alpha \left(x\right)\psi_{3}-\beta \left(x\right)\Delta \psi_{3}\right]}^{}\varphi_{3}\}dx=0 \end{array}$$

for all $\varphi \in C_{0}^{\infty }\left(\bar{\Omega },H\right)$. The Du Bois Reymond variational lemma [61] used for (A3) implies

$$\alpha \left(x\right)\psi_{l}-\beta \left(x\right)\Delta \psi_{l}=0\;\mathrm{for}\;l=0,1,2,3.$$

In consequence, ψ must be a solution of the differential equation

(A4) α(x)ψ−β(x)Δψ=0.
